# Editorial: Innovations and challenges in surgical education

**DOI:** 10.3389/fsurg.2026.1785280

**Published:** 2026-02-10

**Authors:** Gabriel Sandblom, Marco Scarci, Gaetano Gallo, Stavros K. Kakkos, Philipp Taussky, Stefano Cianci

**Affiliations:** 1 Department of Clinical Science and Education Södersjukhuset, Karolinska Institutet, Stockholm, Sweden; 2 National Heart and Lung Institute, Imperial College London, London, United Kingdom; 3 Vita-Salute San Raffaele University, Milan, Italy; 4 University of Patras, Patras, Greece; 5 Beth Israel Deaconess Medical Center and Harvard Medical School, Boston, MA, United States; 6 University of Messina, Messina, Italy

**Keywords:** dexterity, silicone, simulation, skills training, surgery training, telemedecine, tutoring

“The mediocre teacher tells. The good teacher explains. The superior teacher demonstrates. The great teacher inspires.” — William Arthur Ward

Teaching a handicraft is a great challenge, regardless of guild. The master's experience may be transmitted to the disciple verbally, but true learning begins only when the disciple transforms the master's words and example into something self-made. The quality of the outcome may vary at the outset, but with increasing experience, the disciple will eventually acquire sufficient skill to become a reliable craftsman. In the context of teaching surgical skills, this process becomes a very complex balancing act: on the one hand, insufficient quality is unacceptable; on the other, allowing independent training of the skill is crucial for progress. Various attempts have been made to bridge the initial portion of the learning curve for surgical residents without compromising patient safety.

In the United States, as in many other Western countries, there is a shortage of vascular surgeons and a need to increase the number of vascular surgery trainees (Lavanga et al.). The aging population, the increasing prevalence of vascular diseases, and the development of advanced techniques are fuelling the need for more vascular surgeons. Attracting, recruiting, and retaining the essential workforce have been identified as crucial parts of the strategy to cover this need. The skills required in vascular surgery should ideally be acquired at an early age. Still, relatively few undergraduate students are exposed to vascular surgery, and most have little understanding of what it actually means to be a vascular surgeon. In the US, trainees may either enrol in a two-year clinical fellowship after completion of general surgery training or pursue a five-year vascular surgery integrated residency program after medical school, without prior general surgery training. These two training paths in vascular surgery reflect different views on how vascular surgery skills should be taught and on the need for comprehensive surgical training. Regardless of how trainees are recruited, the workforce shortage remains a significant challenge.

Patient safety in surgery not only requires practical skills but also the ability to collaborate with the surgical team. Many situations in cardiovascular surgery require interdisciplinary approaches, including management of lung cancer surgery, complex robotic operations, and postoperative monitoring (Lampridis et al.). The integration of interprofessional education in cardiothoracic training programs varies, but simulation-based training has gained increasing recognition as a way of improving team performance, crisis management, and patient safety. The same probably applies to other surgical disciplines.

In critical situations and when the healthcare system's infrastructure is strained, traditional teaching methods become difficult. This became a reality to manage during the COVID-19 pandemic (1–3). Telemedicine, virtual courses, and increased reliance on virtual simulation training are among the strategies employed to address the challenges encountered in surgical education during the epidemic. Although these teaching methods cannot fully replace real-life training and have elicited varying levels of satisfaction among trainees, they have remained part of the teaching agenda even after the epidemic. Future development of teaching should focus on integrating new virtual methods with real-life skills training.

Training in simulated environments may be a safe way to improve surgical dexterity, but it requires reliable feedback to be effective. Supervision and feedback from an experienced surgeon may enhance skill acquisition, but are subject to rater bias, inconsistent standards, and inefficiency. Repetitive practice of fundamental skills, such as laparoscopic knot-tying, is crucial for developing dexterity but should ideally be continuously evaluated to be effective. Objective and automated evaluations have been used to quantify hand gestures, eye movements, or instrument handling. Reliability Volume is an approach that involves registering the instrument trajectory to account for spatial accuracy and performance consistency across multiple sessions (Yu et al.). Reliability is the probability of completing the task in a virtual simulated environment under specified conditions, and Volume denotes the corresponding workspace volume. Reliability Volume provides feedback that is not subject to human-assessor bias and still offers a dynamic, comprehensive, and practice-oriented assessment framework defined by a standard established by an experienced surgeon.

Artificial intelligence has also been used to track hand motions and enhance surgical training. Economic and precise hand motions are signs of dexterity that may be assessed using sensors in simulated environments. By integrating artificial intelligence algorithms into hand and instrument tracking, more effective and targeted feedback is provided. Machine learning, including supervised, unsupervised, reinforced, and deep learning, offers distinct training paradigms (Yangi et al.). These are relatively new tools in surgical education, but advances in machine-learning methods are expected.

Virtual simulated environments offer many advantages but cannot completely replace physical models. Hernia repair and appendectomy are some of the most common surgical procedures and are among those that surgical trainees are expected to master early in their training. There are, however, no good models for hernia surgery training, and those used for appendectomy have limitations. Single-use synthetic training models for appendectomy training are commercially available, but they are costly and not accessible to all trainees. However, silicon is relatively cheap and suitable for creating appendicitis models. Because silicone can be varied in stiffness and colour, it can simulate different tissues. In a validation study, a silicone model designed for laparoscopic appendectomy simulation effectively enabled residents to perform most critical procedural steps (Roche et al.).

The methods for teaching surgery will likely continue to evolve, incorporating new technologies for effective feedback while also addressing the need to adapt these methods to emerging surgical techniques. Effective teaching that does not compromise patient safety is crucial not only for tertiary centres and centres of excellence, but also for the community at large and for healthcare systems with limited resources.
